# Detection of Human Papilloma Virus (HPV) in oral mucosa of women with cervical lesions and their relation to oral sex practices

**DOI:** 10.1186/1750-9378-5-25

**Published:** 2010-12-04

**Authors:** Luis O Sánchez-Vargas, Cecilia Díaz-Hernández, Alejandro Martinez-Martinez

**Affiliations:** 1 Instituto de Ciencias Biomédicas, Universidad Autónoma de Ciudad Juárez, Ciudad Juárez, Chihuahua, México; 2 Clínica de Displasias. Jurisdicción Sanitaria No. II. Servicios de Salud de CHIH. Paseo Triunfo de la Republica No. 3530, Ciudad Juárez, CHIH Ciudad Juárez, Chihuahua, México

## Abstract

**Background:**

Previous studies have either investigated the relationship of HPV with oral cancer or the prevalence of HPV on the oral cavity. The purpose of this investigation was to study the prevalence of HPV in oral cavity of women with oral sex practices and cervical lesions.

**Methods:**

Forty six (46) non-smokers and non-alcoholic patients attended the "Clínica de Displasias" of "Ciudad Juarez" were sampled. This population had a CIN diagnosis sometime between the previous six months. On previous consent they filled out a questionnaire related to their oral sex practices. Afterwards one swab from cheeks and another from palate/gum were taken; PCR was used to determine generic HPV, HPV16 and HPV18.

**Results:**

Seventy two percent (72%) of the patients stated to have oral sex practices regularly which all of them were positive to HPV either in oral mucus, palate/gum or both. The total of the given results showed that 35% had HPV16; among those distributed in 26% with regular oral sex practices and 9% stated as never practiced oral sex. An association was found between oral HPV16 positivity and progression to cervical CIN advanced lesions. On the other hand HPV18 was not detected. The frequency of HPV16 was higher in buccal mucosa (23%) versus palate/gum (16%).

**Conclusions:**

This study suggests that buccal HPV16 infection is associated with CIN progression.

## Background

Human papillomaviruses (HPVs) are a family of small (55 nm) icosahedral, non-enveloped virus with a circular double-stranded DNA genome of 7-8 kbp and with a special affinity for epithelial cells [1,2]. Over 200 genotypes of papillomaviruses infect the skin and mucosal surfaces [2]. The most common oncogenic HPV are associated with leukoplakia and squamous carcinoma. While the majority of the HPV types have affinity to grow on skin, oral lesions, genitals, anal and larynx [1]. Some HPV types are considered as high risk; most notably 16, 18, 31, 33, 35, 39, 45, 52, and 58 and have been shown to be a necessary cause for Cervical Cancer development [3]. Cervical cancer is a major public health problem around the world; in some developing countries it is the most frequent female cancer, as well as the main cause of cancer related death among women [4,5].

The potentially oncogenic HPVs has been associated with oral squamous cell carcinoma [6,7]. Some evidence has linked them to orogenital contact with the transmission of papillomavirus from the genital zone to the oral cavity [8]. It is suggested that oral HPV infection frequency is different from cervical infection and associated with age [9]. Other studies have detected the presence of HPV in the epithelium of the oropharynx in women with genital HPV by using a cytological examination and the Papanicolaou technique [10]. These studies alert the possibility of a natural reservoir of HPV at a locus outside of the genital region, which could serve as a reinfection focus [10]. Additionally the HPV is rarely present in the vagina of virgin women even with the use of tampons or digital penetration [11].

Despite the recognition of a HPV-associated oral malignancy, it is unclear to what extent cervical HPV infection is translocated to oral HPV infection. Previous studies suggested that oral HPV infection analogously to cervical infection is associated with sexual behavior and immunosuppressant [12,13]. The majority of the studies of buccal HPVs made in the past have explored the relationship between HPV and the development of oral cancer; these studies detected HPV in DNA extracted from the oral cavity of patients with oral lesions and/or abnormalities [14-18] shown that HPVs that infect the genital area can also infect the oral cavity [19]. In healthy Japanese; HPV in oral cavity was present in 0.6% of the population [20]. Other studies have proposed that mothers may serve as the source of infant HPV infection which suggested the possibility of a non-sexual transmission of the virus [21]. In the few studies in which oral and anogenital HPV infections were analyzed, oral HPV infection frequency appeared to be lower than anogenital infection [16,17,22,23]. However, oral and cervical HPV prevalence were similar in a small group with high prevalence of oral or anogenital condylomata [16]. These studies were performed in a high risk population therefore the relationship between HPV infection in cervix and oral cavity related to oral sex practices remains unexplored. The aim of this study was to determine the frequency of HPV in the oral cavity of a Mexican women population with histopathological diagnosis of cervical lesions and to describe the viral infection in relation to oral sex practices and habits to share personal objects such as toothbrush.

## Results

This study included 46 voluntary non-smoker and non-alcoholic women that attend to the "Clínica de Displasias del Sector Salud" in the City of Juarez, México. The female subjects attend to this clinic because they presented previous CIN alterations. After they filled out the inform consent and the questionnaire; the patients were instructed by imitation for autosampling the oral cavity. Once in the laboratory and after DNA extraction the human β-globin gene was amplified as an internal control of human DNA as well as a DNA with quality for PCR. Those cases that failed in the β-globin gene amplification were excluded rather than those samples tested positive for β-globin were further used for generic HPV that amplify a wide range of HPV types. After generic HPV positivity the specific PCR for HPV16 and HPV18 were performed. Two regions were sampled in the oral cavity; the first one was the buccal mucosa and the second one was the palate and gum altogether (P/G). All women studied carried generic HPV either in mucosa or P/G, this gives a frequency of 100% for the buccal cavity. However, if the mucosa and P/G are considered separately (Table 1) the percentages of HPV infection were as follows: for generic HPV in the buccal mucosa was 86%, and for P/G was 88% and for HPV16 23% for mucosa and 16% for P/G. The higher frequency of HPV16 in woman having oral sex would suggest a higher risk, however number are too small to see any significant risk.

**Table 1:** Frequency of HPV by buccal region and its relation with oral sex practices

Overall (N = 43)	Oral sex	Generic HPV		HPV16	
	
Buccal mucosa		n	(%)	n	(%)
	No	11	26	3	7
	Yes	26	60	7	16
		
	Total	37	86	10	23
					

Palate/gum		n	(%)	n	(%)
	No	9	21		2
	Yes	29	67	6	14
		
	Total	38	88	7	16

Sixty-three percent (63%) of the studied female subjects were married, 19% were in common law marriage and 7% were singles. The mean age was 35 years old ranging between 19 to 63 years old (not shown in tables). Table 2 shows the results of the applied questionnaire. Generic HPV was detected in all cases either in palate-gum, buccal mucosa, or both. However, HPV16 was detected in 35% of all patients and 73% of the patients stated to practice oral sex frequently. The association between the presence of HPV16 and the frequently practice of oral sex was not observed. From the total women, 53% had oral sex mutually (fellatio and cunnilingus, Table 2). This group had the highest frequency of HPV16 (53%) among those patients stated to practice oral sex. Interestingly the only three women that practice fellatio but did not received cunnilingus (7%) were generic HPV positive but HPV16 negative.

**Table 2:** Buccal HPV16 associated to oral sex practices

	Overall (N = 43)		HPV16 negative (N = 28)		HPV16 positive (N = 15)	
	n	%	n	%	n	%
**Oral Sex frequency**						
Never	12	28	8	29	4	27
Frequently	31	72	20	71	11	73

**Oral Sex type**						
Do not practice	1	2	1	4	0	0
To her partner	3	7	3	11	0	0
Both	23	53	15	54	8	53
Did not answer	16	37	9	32	7	47

*Histological biopsy results						
Without alterations	9	21	7	25	2	13
Inflammatory Alterations	12	28	9	32	3	20
CIN-I & CIN-II	22	51	12	43	10	67

**Use of condom while practicing oral sex**						
Do not practice oral sex	12	28	8	29	4	27
Use of Condom	1	2	1	4	0	0
Do not use Condom	25	58	16	57	9	60
Did not answer	5	12	3	11	2	13

**Number of Partners to whom practice oral sex**						
Do not practice oral sex	1	2	1	4	0	0
One	20	47	12	43	8	53
Two	10	23	7	25	3	20
More than two	1	2	1	4	0	0
Did not answer	11	26	7	25	4	27

**Personal objects sharing**						
Do not practice	23	53	17	61	6	40
Occasionally	17	40	9	32	8	53
Frequently	2	5	1	4	1	7
Did not answer	1	2	1	4	0	0

* Mann Whitney U = 1.9819 p = 0.0237

[42]; http://department.obg.cuhk.edu.hk/researchsupport/MannWhitney.ASP).

All women were CIN diagnosed along the previous six months (according to Bethesda classification) because of that; it is clearly evident the association between oral HPV16 positivity and CIN progression 51% (Mann Whitney U test, p = 0.023), followed by 28% with inflammatory alterations; and finally 21% did not present cervical alterations (Table 2). Two women that evolved positively to the treatment at that time of gynecological visit were observed. They had no cervical alterations but were HPV16 positive.

The majority of the patients stated not to use condoms while practicing oral sex, and 60% of them were buccal HPV16 positive. Apparently the use of condoms during oral sex would prevent infection of the oral mucosa (Table 2).

The biggest group of patients (47%) declared to have only one sex partner, 23% had two partners and 2% had more than two partners (Table 2).

The majority (53%) of the women stated not to share spoons, toothbrush or candies (Table 2). But an important fraction (40%) recognized to share those objects occasionally (Table 2).

## Discussion

The prevalence rate of HPV in normal oral mucosa has been reported to vary greatly because of differences in types of samples, collection, detection methods, level of sensitivity, PCR primers used, and PCR inhibitors [10,15,22-32]. A previous study showed a high prevalence of oral HPV infection (81%) among healthy people in Japan [20].

In this study, we performed the HPV detection by PCR using the MY09/MY11 primer pair; which, it is widely used on epidemiological studies and showed to be equally sensitive than confirmatory nested PCR with GP5+/GP6+ primers, with a correlation of 94% [33-35]. Generic HPV in our sampled population had a frequency of 100%. These women that attend to the "Clinica de Displasias de Ciudad Juárez" can be considered as high risk population since they came to this clinic attending some cervical abnormality. It is notably interesting that all these women were positive and taking into account that they had some cervical problem; this number became comparable to the 81% found in oral cavity of healthy Japanese and the 90% buccal HPV among positive genital HPV in Brazilian women [7,20]. Buccal presence of HPV16 in our study was 35% (Table 2). However, we have observed differences by the anatomic region of the oral cavity sampled that was 23% for buccal mucosa to 16% on P/G. To sum up, this HPV point prevalence was higher than those of other normal oral HPV studies as well as in high risk population [20,26,30]. The reasons of such a high HPV prevalence in the normal oral cavity in women of the City of Juarez are not yet explored. Further studies are needed to clarify this as well as the differences in HPV infection at each normal oral site and the use of condoms along with preventive vaccination as a strategy to prevent infections of the oral mucosa.

We evaluated HPVs only in buccal samples. Although in cancers of the upper aerodigestive tract HPV has been detected predominantly in the oropharynx and tonsil [29,35]. A risk factor for the occurrence of HPV-infected oral and for presence of HPV in the normal cervix is an increase in the number of sexual partners [10,20,28], but in our study the number of sex partners did not seem to increase the risk of HPV-16 oral infection. Oral sex practices may affect oral and cervical sites similarly. Local factors that have been found to influence the persistence of HPV at the cervix may affect the natural history of oral HPV infections. These factors include coinfection by *Chlamydia trachomatis *[30,31] or herpes simplex virus [32], smoking [36], age [37], HPV type [38], and the use of hormones and contraceptives [23,36,39]. However, all these reports did not implement any questionnaire to identify any factors behind the transmission of HPVs. We have collected information about sexual behavior of the patients, to examine the relationship between cunnilingus and incidence of mouth HPV infection.

In our study HPV16 in mouth was significantly associated with genital CIN progression (Mann Whitney U test, p = 0.023), suggesting that women with HPV16 persistent infections and progression to advanced genital lesions have higher risk of HPV16 detection in the oral mucosa. We found HPV16 infection in a media of 37 years. As this type of HPV is related to cancer; it is also considered as high-risk mucosal type virus [40] and this phenomena should be known by the dentists and pathologists.

The role of men as possible vectors of HPV has been discussed previously [41]. Our results suggest that transmission of HPV occurs not only via sexual contact but also through oral contact. The fact that women that stated not to receive cunnilingus were HPV16 free; this probably reflects that their partner is not translocating the virus from the women genitals to the mouth but probably the autoinoculation is more frequent that we think.

In a cross-sectional study of Fahkyr [23], oral HPV infection was found to be less prevalent than cervical HPV infection in both HIV-positive and -negative women. Oral HPV infections were detected in approximately 25% of HIV-positive women and 9% of HIV-negative women. Our results showed a much higher prevalence in oral cavity of women with or without cervical lesions then the study of Fahkyr [23]. These data suggest that the oral cavity may be a reservoir of HPV infection with a sufficiently high prevalence to affect the dynamics of HPV transmission between the populations. Several aspects for the relationship among cervical- and oral-HPV remain poorly described. A prospective study to clarify the interrelationship between HPV infections at both sites and to understand possible differences in incidence and factors affecting clearance or persistence in the oral cavity and the cervix is warranted. These include differences by anatomic site in HPV prevalence and HPV type distribution. Also unknown are the prevalence of single and multiple oral or cervical infections and whether multiple infections are type concordant.

Our data hint, it is that oral cavity should be considered as a potential reservoir for HPV and may not be entirely independent of the cervical reservoir as well as the partner genitalia. In this study we did not sample the partner's genitalia but we assume that both genitalia are infected since these are considered as sexually transmissible infections. Thus the presence of the virus in the oral cavity should be explained not only by oral contact with genitalia but also by autoinoculation. We are aware about the different prevalence data in oral HPV DNA detection ought to different populations and methodologies. Consequently comparative studies will be required in parallel studies of the relationship between incident squamous epithelial lesions and persistence oral HPV. This will help to understand the involvement of HPV in the development of oral cancer as well as the role of oral cavity in the cervical infection.

## Conclusions

In conclusion, this study suggests that women with genital HPV infection have also some kind of HPV infecting their oral cavity and HPV16 detection in the mouth is associated to HPV16 persistence in the genital tract and CIN progression.

## Methods

### Patients and sampling

This study was performed on summer 2008. All women had a previous CIN diagnosis between six months previous to the study. The Inclusion criteria were any women that attend to "Clínica de Displasias" for any cervical associated problem, no matter their origin or place of living (Mexico or USA), sign an informed consent and fill a questionnaire about their sex habits. The exclusion criteria were: smoking and alcoholism habits. All women studied were above eighteen years old. The full protocol was approved by the Ethics Committee of the Universidad Autonoma de Ciudad Juarez and the Clinica de Displasias. Women were asked to autosampling with a cotton swab the two cheeks and with other cotton swab the palate/gum (P/G). In both cases tissues were rubbed for a minute. Cotton swabs were immersed into 15 mL tube containing 1 mL of transporting media (10 mM Trizma, pH 8.8; 1 mM EDTA; 0.01% Sodium azide; 50 μg/mL Ampicillin; 1 μg/mL Proteinase K) and stored at -20°C in between 24 h after collection.

### DNA extraction and PCR for HPV

Samples were unfreeze, centrifuged at 3,500 rpm in a clinical centrifuge, cotton swab removed and the transporting media was transferred to a 2 mL microtube that was centrifuged 5 min at 3,500 rpm at 4°C. 300 μL of clear supernatant were taken into a new tube and 25 μL of 5 M sodium acetate and 1 mL of isopropanol were added consecutively and centrifuged at 14,000 rpm at 4°C for 5 min. Pellet were washed with 1 mL of 70% ethanol and dried overnight at room temperature. Pellet was dissolved with 100 μL rehydration solution (Promega A7963) and incubated at 65°C for 20 min.

PCRs for generic HPVs were assembled with 5 μL DNA, 12.5 μL 2X GoTaq Green Master Mix (Promega). 5 μL MY11/MY09 primer mix at 2.5 μM each, and 2.5 μL water. PCR conditions were 40 cycles at 94°C for 60 s, 55°C for 60 s, 72°C for 60 s, with an initial denaturation at 94°C for 5 min and a final extension of 72°C for 7 min [33]. PCR products were examined in 2% agarose gels using base pairs standard (Promega G7521). PC04 and GH20 primers for human betha-globin gene were used as internal controls. For HPV16 and HPV18 specific primers were used as described elsewhere [34,35].

### Statistical analysis

Questionnaire answers, previous histopathological results and buccal HPVs results were analyzed with SPSS statistical software version 11 and Mann Whitney U Test [42].

## Competing interests

There are no conflicts of interest by any of the authors involved neither in this study publication nor for the previous 2 years.

## Authors' contributions

LS carried out the molecular genetic studies, participated in the sampling, and questionnaire design, analysis and drafted the manuscript. CD carried out the selection of patients, sampling, questionnaire application, and analysis of data. AM conceived of the study, and participated in its design, statistical analysis, writing and coordination of the study. All authors read and approved the final manuscript.
